# Surface relief formation with light possessing multiple vortices

**DOI:** 10.1515/nanoph-2025-0387

**Published:** 2025-11-18

**Authors:** Junjie Zhao, Kazuro Kizaki, Atsushi Taguchi, Madoka Ono, Soki Hirayama, Takashige Omatsu

**Affiliations:** Department of Applied Physics, Graduate School of Engineering, Tohoku University, Sendai, Japan; Research Institute for Electronic Science, Hokkaido University, Sapporo, Japan; Molecular Chirality Research Center, 12737 Chiba University, Chiba, Japan; Graduate School of Science and Engineering, and Molecular Chirality Research Center, 12737 Chiba University, Chiba, Japan; Department of Electrophysics, National Yang Ming Chiao Tung University, Hsinchu, Taiwan

**Keywords:** optical vortices, surface relief, orbital angular momentum, spin angular momentum

## Abstract

We report the first demonstration of surface relief formation by irradiating material with non-degenerate hybrid vortex modes. These modes are formed via the coherent superposition of two Laguerre–Gaussian (LG) modes with different orbital angular momentum (OAM) indices, and they carry non-zero OAM. Intriguingly, the spatially localized vortex fields, which are associated with multiple phase singularities of the non-degenerate hybrid vortex modes and spin angular momentum (SAM) of circular polarization, can be visualized as fist-like protrusions produced within the fabricated surface relief structures. This demonstration offers new insights into fundamental light–matter interactions via SAM-OAM coupling effects and opens the door to a deeper understanding of the mechanisms underlying the formation of vortex lattices and vortex–antivortex pairs in condensed matter physics. This demonstration also provides a method for fabricating chiral surface relief structures with an odd number of spiral arms, which may be utilized in advanced optical data storage and chiral metasurface applications.

## 1 Introduction

Laguerre–Gaussian (LG) modes are perhaps the most commonly applied examples of optical vortices. They have a ring-shaped annular spatial intensity profile with a central dark core, and carry an orbital angular momentum (OAM) (characterized by an azimuthal index *ℓ* called the topological charge) which is associated with their helical wavefront with an on-axis phase singularity [1], [2], [3]. Also, circularly polarized light carries a spin angular momentum (SAM, characterized by an index *s* = ±1), which originates from its helical electric field [4], [5].

Polymers containing azobenzene (azo-polymers) exhibit a unique photo-induced mass transport characteristic wherein an illuminated azo-polymer moves from a bright region toward a dark region under visible light illumination through the *trans*-*cis* photo-induced isomerization process. This enables the formation of unique surface relief structures which may find application in rewritable engineered optical materials and devices [6], [7], [8], [9], [10], [11]. The resulting surface relief structures typically reflect the spatial intensity and polarization profiles of the irradiating light field [12], [13], [14], [15].

In recent years, it has been discovered that the OAM of an irradiating optical vortex beam twists azo-polymers to fabricate single-armed chiral surface relief structures on the azo-polymer film with the assistance of SAM [16], [17], [18]. This is a manifestation of the direct imprint of the helicities (helical wavefront) of a light field. Also, spiral surface relief formation has been demonstrated even through linearly polarized higher-order optical vortex irradiation under tightly focused condition owing to interference between longitudinal and transverse optical fields [13]. Furthermore, the formation of surface relief structures with 2 $\left|\ell \right|$ spiral arms, which occurs between the rotating optical field induced mass transport and the SAM of an irradiating optical field, have been demonstrated by employing topological-charge degenerate hybrid vortex modes (referred here as degenerate hybrid vortex modes) with 2 $\left|\ell \right|$ petal like bright spots and zero OAM, formed of the coherent superposition of positive and negative *ℓ* ^th^ order LG modes with ±*ℓ* [19]. These spiral surface reliefs will potentially play as chiral engineered materials, which cannot be superimposed onto their mirror image, to demonstrate the detection and generation of helical optical fields on a nanoscale [20].

A topological-charge non-degenerate hybrid vortex mode (referred here as non-degenerate hybrid vortex modes) is formed via the coherent superposition of two LG modes with different OAM indices (*ℓ* and *ℓ*′; *ℓ* ≠ −*ℓ*′), and it possesses space-invariant beam propagation properties. It notably has multiple vortices possessing non-zero OAM, associated with multiple singularities, and $\left|\ell -\ell^{\prime }\right|$ petal-like bright spots [21], [22], [23], while degenerate hybrid vortex modes carry zero OAM. Non-degenerate hybrid vortex modes will locally twist azo-polymers into more complex surface structures, such as surface reliefs with multiple spiral arms or vortices, which are a consequence of their multiple vortices. Note that these degenerate/non-degenerate hybrid vortex modes are different from frequency-degenerated vortex modes in laser cavities [24], [25].

Multiple vortices (or multiple singularities) have been observed in condensed matter, including superfluid and superconductors [26], [27], and they have been intensely studied, notably regarding the formation of vortex lattices and vortex-antivortex pairs [28], [29], [30], [31]. The multiple vortices of a non-degenerate hybrid vortex mode may prove useful in such studies and offer new insights into fundamental light–matter interactions as an analogous optical counterpart of multiple vortices in condensed matter physics. For instance, the creation and annihilation of exotic vortex structures, such as vortex lattices and vortex-antivortex pairs, in materials. However, notably, to date, all demonstrations of optical vortex-induced structured reliefs have been performed by using optical vortices with a single phase-singularity, and there are few reports detailing the simultaneous interaction between non-degenerate hybrid vortex modes with multiple vortices and materials.

In this work, we report on the first demonstration of surface relief formation in azo-polymer utilizing illumination by non-degenerate hybrid vortex modes with $\left|\ell \right|$ – $\left|\ell^{\prime }\right|$ = 1, to ensure sufficient spatial overlap between two LG modes with different azimuthal indices. These modes are herein referred to as *ℓ*th order non-degenerate hybrid vortex modes. Interestingly, the spatially localized vortices of these non-degenerate hybrid vortex modes are directly imprinted (visualized) as fist-like protrusions within the fabricated surface relief structures. Furthermore, we demonstrate the first fabrication of exotic chiral surface relief structures with an odd (≥3) number of curved arms, even without the help of temporal rotation, while the degenerate hybrid vortex modes allow only the formation of surface relief structures with 2 $\left|\ell \right|$ spiral arms with the assistance of temporal rotation [19].

## 2 Experiments

### 2.1 Non-degenerate hybrid vortex mode

The LG_*ℓ*_ modes, with a radial index *p* = 0 and an azimuthal index *ℓ*, *i.e.*, topological charge, at *z* = 0, can be expressed as follows:

(1) $$\text{L}{\text{G}}_{\ell }\left(r,\phi \right)\propto {\left(\frac{r}{\omega_{0}}\right)}^{\left|\ell \right|}\exp\left(-\frac{r^{2}}{\omega_{0}^{2}}\right)\exp\left(i\ell \phi \right)$$

where *r* and *ϕ* are the radial and azimuthal coordinates, and *ω*_0_ is the beam radius, respectively. The coherent superposition of two orthogonal LG_*ℓ*_ and $\text{L}{\text{G}}_{\ell^{\prime }}$ modes form a flower-shaped beam with an odd number of petals, given by

(2) $$\begin{array}{ll} & \text{L}{\text{G}}_{\ell }\left(r,\phi \right)\cos\left(\frac{\theta }{2}\right){\text{e}}^{\text{i}\frac{\beta }{2}}+\text{L}{\text{G}}_{\ell^{\prime }}\left(r,\phi \right)\sin\left(\frac{\theta }{2}\right){\text{e}}^{-\text{i}\frac{\beta }{2}}\\ & \;\propto {\left(\frac{r}{\omega_{0}}\right)}^{\left|\ell^{\prime }\right|}\exp\left(-\frac{r^{2}}{\omega_{0}^{2}}\right)\exp\left(i\left(-\frac{1}{2}\right)\phi \right)\\ & \;\;\left[\left(\frac{r}{\omega_{0}}\right)\cos\left(\frac{\theta }{2}\right)\cdot \exp\left(i\left(\ell +\frac{1}{2}\right)\phi \right)\right.\\ & \;\;\left.+\sin\left(\frac{\theta }{2}\right)\cdot \exp\left(-i\left(\ell +\frac{1}{2}\right)\phi \right)\right] \end{array}$$

where *θ* is the ellipticity, and *β* is the initial relative phase, respectively. The intensity profile $I\left(r,\phi \right)$ of the petal beam with *θ* = *π*/2 is as follows:

(3) $$\begin{array}{ll} I\left(r,\phi \right) & \propto {\left(\frac{r}{\omega_{0}}\right)}^{2\left|\ell^{\prime }\right|}\exp\left(-\frac{2r^{2}}{\omega_{0}^{2}}\right)\left[1+{\left(\frac{r}{\omega_{0}}\right)}^{2}\right.\\ & \;\left.+2\left(\frac{r}{\omega_{0}}\right)\cos\left(\left(2\ell -1\right)\phi \right)\right] \end{array}$$

Here *r* and *ϕ* are the radial and azimuthal coordinates. These non-degenerate hybrid vortex modes with circular polarization exhibit an azimuthally modulated spatial form with an odd number of petals, and multiple local vortices associated with multiple phase singularities.

The time-averaged optical scattering force in air <**F**(*x*, *y*)> is given by.

(4) $$\left\langle F\right\rangle \propto \text{Im}\left(E\times B^{*}\right)$$

where **E**(*x*, *y*) (=(*E*_*x*_, *E*_*y*_, *E*_*z*_)) is the optical field of the hybrid vortex mode, which can be numerically simulated by using conventional finite-difference time-domain (FDTD) methods [30].

Also, the canonical (orbital) **P**, spin, and Poynting **Π** momentum densities are expressed as follows:

(5) $$P=\frac{1}{2\omega }\text{Im}\left[E^{*}\cdot \nabla E+H^{*}\cdot \nabla H\right]≃\frac{1}{\omega }\text{Im}\left[E^{*}\cdot \nabla E\right]$$

(6) $$\Pi =c^{-1}\text{Re}\left(E^{*}\times H\right)$$

**Figure 1:**
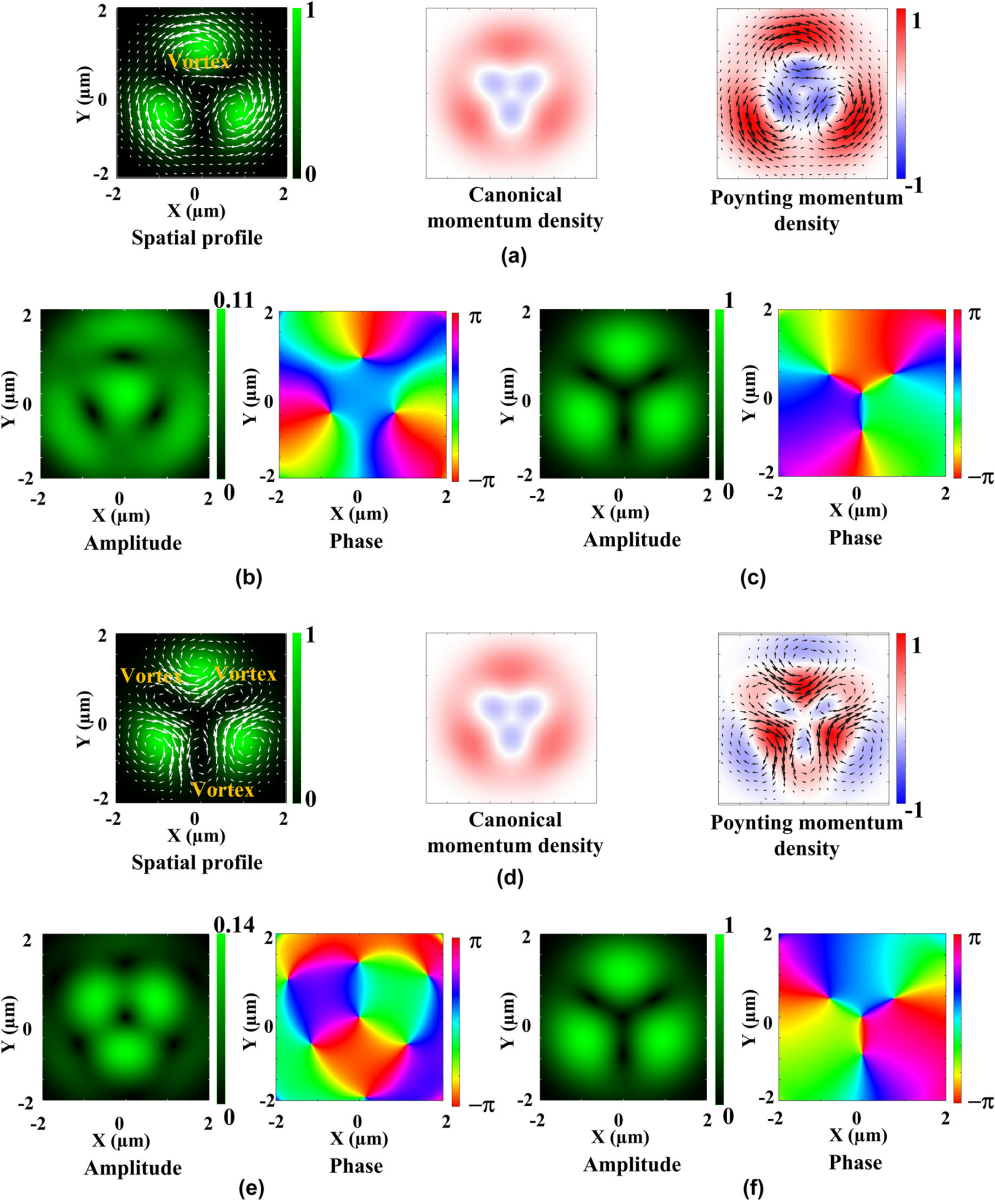
Theoretically modelled spatial forms, time-averaged transverse scattering forces, canonical (orbital) and Poynting momentum densities of 2^nd^ order non-degenerate hybrid modes. (a) Theoretically modelled spatial forms, time-averaged transverse scattering forces, canonical (orbital) and Poynting momentum densities (in air), (b) longitudinal electric field (amplitude and phase), and (c) transverse electric field (amplitude and phase) of 2^nd^ order non-degenerate hybrid modes with 3 petal-like bright spots when *ℓ* and *s* possess the same sign. (d) Theoretically modelled spatial forms, time-averaged transverse scattering forces, canonical (orbital) and Poynting momentum densities (in air), (e) longitudinal electric field (amplitude and phase), and (f) transverse electric field (amplitude and phase) of 2^nd^ order non-degenerate hybrid modes with 3 petal-like bright spots when *ℓ* and *s* possess the opposite sign. Orbital and Poynting momentum densities are normalized to the maximum value. Also, amplitudes of longitudinal and transverse electric fields are normalized to the maximum value. The scale bar in the optical field intensities indicates brightness from maximum (whitish green) to minimum (black). The sign (plus or minus) of momentum densities then indicates counterclockwise or clockwise direction. Scattering force vectors are shown by white (spatial form) and black (Poynting momentum density) arrows. Size of simulation graphs is 4 × 4 µm.

Figure 1 summarizes the spatial form, time-averaged scattering force, azimuthal components of canonical (orbital), and Poynting momentum densities, and longitudinal and transverse electric fields of non-degenerate hybrid vortex modes with *ℓ* = 2 and *ℓ*′ = −1. The sign (plus or minus) of momentum densities then indicates counterclockwise or clockwise direction. It is worth noting that the numerical aperture of the focusing lens was then assumed to be 0.6 to match the experimental conditions. When *ℓ* and *s* have the same sign (*ℓ*′ and *s* have the opposite sign), the non-degenerate hybrid vortex mode produces a single clockwise vortex field around the central dark spot owing to the enhancement of Poynting momentum via constructive coupling between orbital and spin momentum (the bright spot appeared at the center of wheel-shaped longitudinal electric field.) (Figure 1(a) and (b)). Conversely, in the case of *ℓ* and *s* having the opposite sign (*ℓ*′ and *s* have the same sign), such a central clockwise vortex field disappears due to the reduction of Poynting momentum via destructive orbital and spin momentum coupling, and instead, the multiple counterclockwise vortices manifest around the multiple dark nodes (the bright spot then disappeared at the center of longitudinal electric field, and azimuthal 3 petals appeared.) (Figure 1(c) and (d)).

Note that these constructive and destructive orbital and spin momentum coupling effects (the longitudinal electric field amplitude associated with orbital and spin momentum coupling effects is smaller than the transverse electric field amplitude) do not affect significantly the spatial intensity profiles of hybrid vortex modes (as shown in Figure 1(a) and (d)) even during beam propagation (space-invariant properties). Thus, multiple vortices appear around a central dark spot or dark nodes of non-degenerate hybrid vortex modes, but are invisible in physical space (in both the near and far fields).

**Figure 2:**
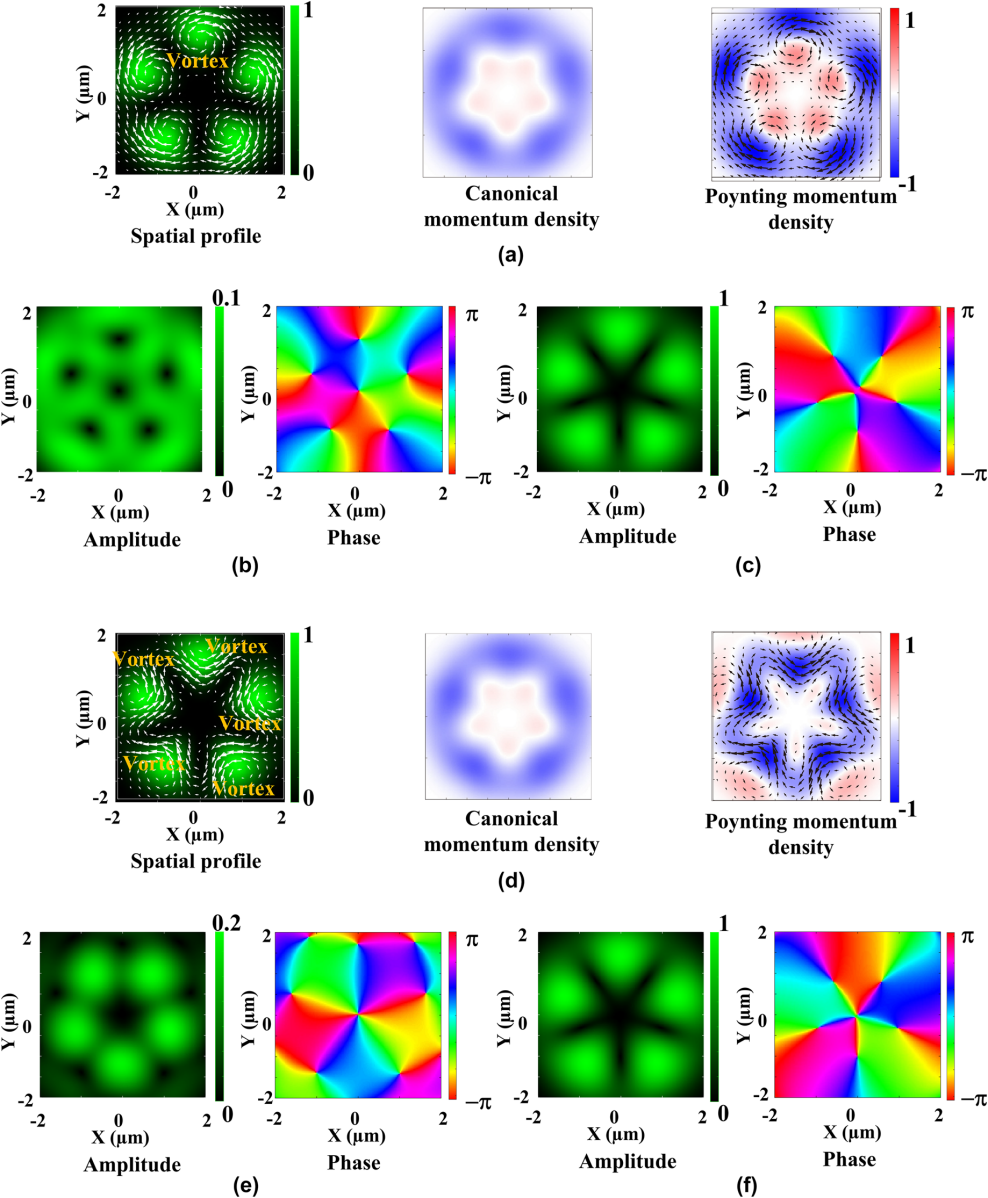
Theoretical modelled spatial forms, time-averaged transverse scattering forces, canonical (orbital) and Poynting momentum densities of 3^rd^ order non-degenerate hybrid modes. (a) Theoretically modelled spatial forms, time-averaged transverse scattering forces, canonical (orbital) and Poynting momentum densities (in air), (b) longitudinal electric field (amplitude and phase), and (c) transverse electric field (amplitude and phase) of 3^rd^ order non-degenerate hybrid modes with 5 petal-like bright spots when *ℓ* and *s* possess the same sign. (d) Theoretically modelled spatial forms, time-averaged transverse scattering forces, canonical (orbital) and Poynting momentum densities (in air), (e) longitudinal electric field (amplitude and phase), and (f) transverse electric field (amplitude and phase) of 3^rd^ order non-degenerate hybrid modes with 5 petal-like bright spots when *ℓ* and *s* possess the opposite sign. Orbital and Poynting momentum densities are normalized to the maximum value. Also, amplitudes of longitudinal and transverse electric fields are normalized to the maximum value. The scale bar in the optical field intensities indicates brightness from maximum (whitish green) to minimum (black). The sign (plus or minus) of momentum densities then indicates counterclockwise or clockwise direction. Scattering force vectors are shown by white (spatial form) and black (Poynting momentum density) arrows. Size of simulation graphs is 4 × 4 µm.

Figure 2 summarizes the spatial form, time-averaged scattering force, azimuthal components of canonical (orbital), and Poynting momentum densities, and longitudinal and transverse electric fields of non-degenerate hybrid vortex modes with *ℓ* = −3 and *ℓ*′ = 2 with 5 petal-like bright spots. When *ℓ* had the same sign as *s*, the non-degenerate hybrid vortex mode produces a counterclockwise inward vortex force towards the central dark spot. In contrast, when *ℓ* and *s* possess the opposite sign, the inward vortex force disappears, and instead, the clockwise vortex forces towards the multiple dark nodes appear.

### 2.2 Methods

Azo-polymer (poly orange tom 1 (POT) polyamine, monomer weight: 484 g/mol; molecular weight: ∼190,000 g/mol; degree of polymerization: ∼400) was used in this experiment [32], [33]. The azo-polymer cyclohexanone solution was dropped and spin-coated on a glass substrate to form a film with a thickness of ∼1 µm, with the measured optical density for 532 nm to be ∼0.34.

**Figure 3:**
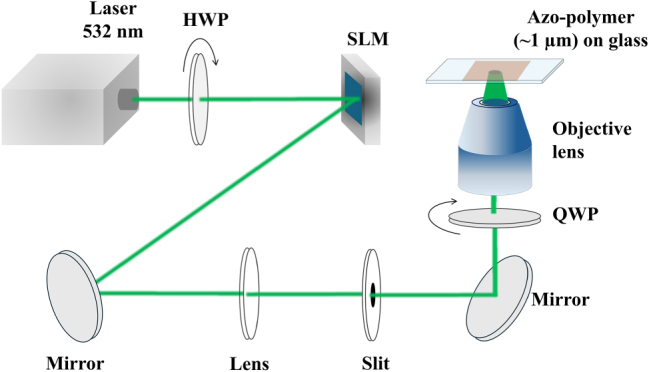
Schematic showing the experimental setup used to fabricate surface relief structures in azo-polymers. HWP: half-wave plate; SLM: spatial light modulator; and QWP: quarter-wave plate.

Figure 3 shows the experimental setup used to fabricate surface relief structures on the azo-polymer film. A continuous-wave (CW) green laser (wavelength: 532 nm) was horizontally polarized along a slow axis (*x*-axis) of a reflective-type spatial light modulator (SLM) (Holoeye, Pluto-2.1; spatial resolution: 1,920 × 1,080 pixels, pixel pitch: 8.0 µm), and its output was incident onto the SLM. This shaped the beam to produce non-degenerate hybrid vortex modes with $\left|\ell -\ell^{\prime }\right|$ petals (formed via the coherent superposition of the *ℓ* ^th^ and *ℓ*′^th^ LG modes), by employing a blazed hologram with encoded spatial amplitude and phase profiles along the *x* direction, so as to modulate both the amplitude and phase of incident laser beam [19]. The generated non-degenerate hybrid vortex mode was then selected as the 1^st^ order diffracted beam by using a lens and a slit, and it was directed onto a microscope imaging system. It is worth mentioning that the non-degenerate hybrid vortex beam was then circularly polarized by using a quarter-wave plate (QWP), so that it also carried SAM. Further, it was focused to be a ∼4 µm diameter spot onto the azo-polymer film by an objective lens (NA = 0.6). The laser power and exposure time (when illuminating the azo-polymer film) were fixed at 15 µW (∼100 W/cm^2^) and 20 s, respectively. When the laser power and/or exposure time exceeded these values, excessive mass-transport or photo-bleaching of the azo-polymer occurred, resulting in distortion of the surface relief structures. The surface relief structures were then characterized using an atomic force microscope SPM-9700 (Shimadzu) with Olympus OMCL-AC240TS-C3 cantilever, which had a spatial resolution of 30 nm.

## 3 Results and discussion

**Figure 4:**
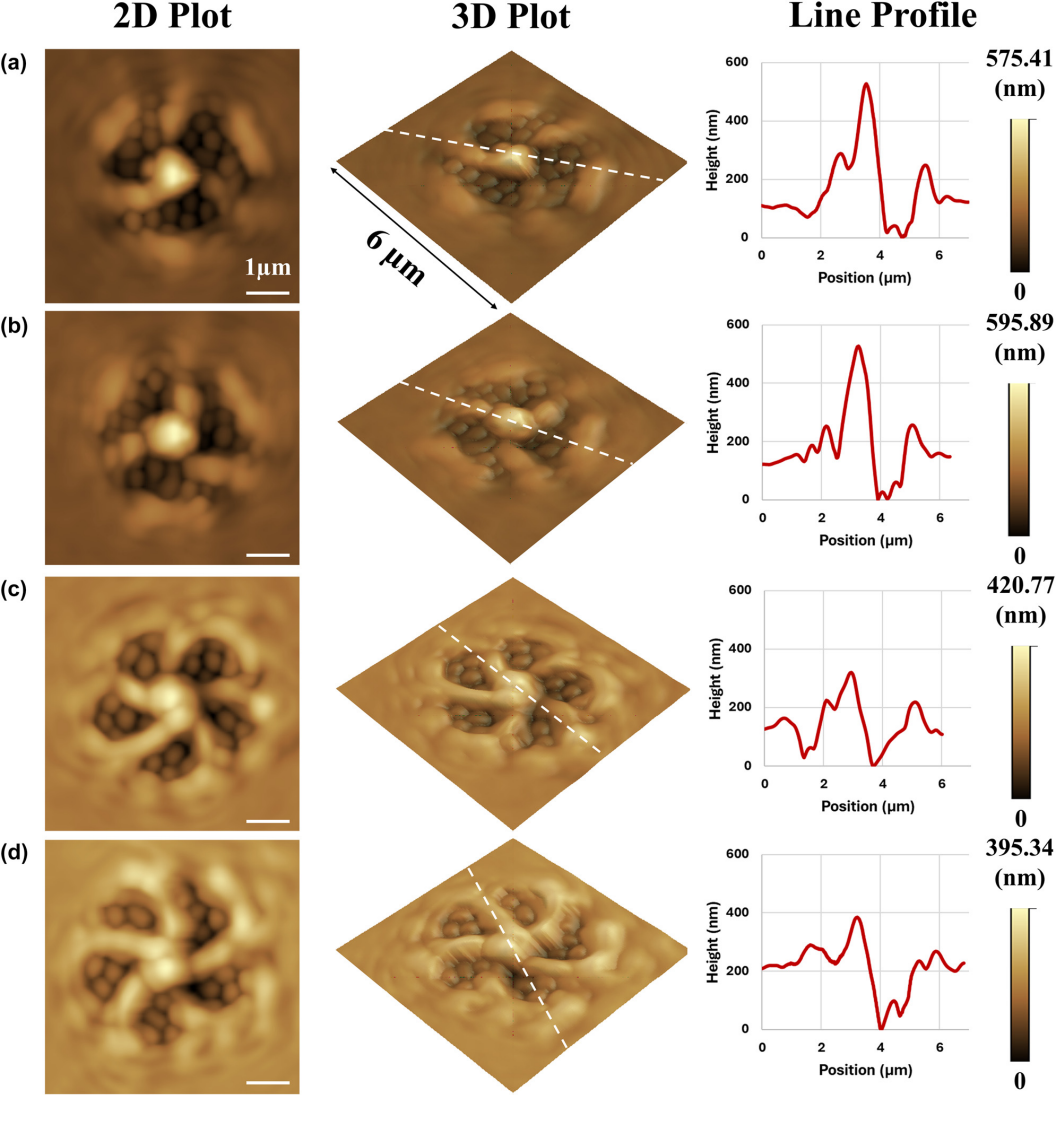
Atomic force microscope images showing the fabricated surface relief structures formed on azo-polymer film under illumination using 2^nd^ order (a, b) and 3^rd^ order (c, d) hybrid vortex modes, where *ℓ* and *s* have the same sign (*ℓ′* and *s* have the opposite sign). White line represents a 1 µm scale bar. Line profiles of surface relief structures were measured along the path indicated by broken white line in the 3D plots.

**Figure 5:**
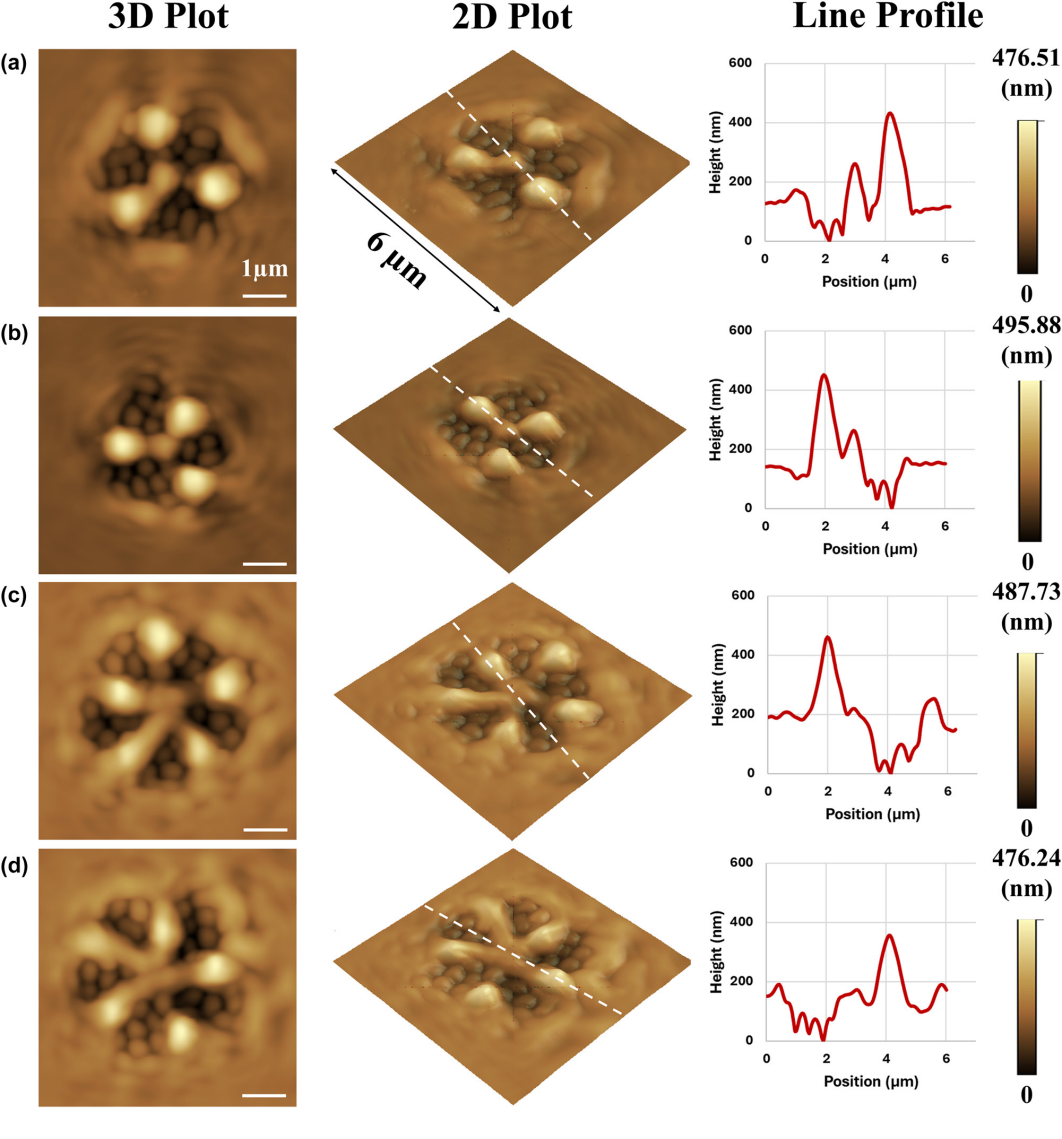
Atomic force microscope images showing the fabricated surface relief structures formed on azo-polymer film under illumination using 2^nd^ order (a, b) and 3^rd^ order (c, d) hybrid vortex modes, where *ℓ* and *s* have the opposite sign (*ℓ′* and *s* have the same sign). White line represents a 1 µm scale bar. Line profiles of surface relief structures were measured along the path indicated by broken white line in the 3D plots.

Figure 4 summarizes the characteristics of the surface relief structures fabricated by the irradiation of 2^nd^ and 3^rd^ order non-degenerate hybrid vortex modes with 3 petals ( $\left|\ell \right|$ = 2, $\left|\ell^{\prime }\right|$ = 1) (Figure 4(a) and (b)) and 5 petals ( $\left|\ell \right|$ = 3, $\left|\ell^{\prime }\right|$ = 2) (Figure 4(c) and (d)). The fabricated surface relief structures typically exhibited a diameter of 4–5 µm and a height of 400–600 nm. Interestingly, whenℓ and *s* had the same sign (*ℓ*′ and *s* had the opposite sign), the structures possessed 3 (or 5) curved arms with a central fist-like protrusion (central core possessing a diameter of 1.5–2 µm and a height of 200–400 nm), these arms manifesting the existence of a vortex field in the central dark spot of the non-degenerate hybrid vortex modes owing to constructive coupling effects between orbital and spin momentum. The handedness of the fabricated surface relief structures could be reversed by simply inverting the sign of all indices, *ℓ*, *ℓ*′, and *s*.

Wherein *ℓ* had the opposite sign of *s* (*ℓ*′ and *s* had the same sign) (Figure 5), the fabricated surface relief structures possessed 3 (or 5) straight (not curved) arms. In this case, fist-like protrusions (fists possessing a diameter of 1 µm and a height of 200–300 nm) around the region of the dark nodes of the irradiating non-degenerate hybrid vortex mode were observed, instead of being located at the central core, due to the disappearance of the vortex field (destructive coupling between orbital and spin momentum) in the center of these non-degenerate hybrid vortex modes. The surface relief structures almost remained unchanged when inverting the sign of the indices, *ℓ*, *ℓ*′, and *s*.

It is worth mentioning that these fabricated surface reliefs in azo-polymer film were rather stable (a lifetime of fabricated surface reliefs was typically 1–2 weeks even in a conventional bright room), unless the reliefs are directly irradiated by spatially uniform blue–green laser with a moderate intensity of ∼100 W/cm^2^ or the reliefs are heated to the glass transition temperature of azo-polymers (∼100 °C) [34]. Further, a positive replica of surface relief can be easily produced by employing nano-imprinting technology based on ultraviolet photo-polymerization in combination with a negative silicon rubber replica [35].

To investigate how the non-degenerate hybrid vortex modes drive the mass transport of the azo-polymers, the non-degenerate hybrid vortex mode-induced time-averaged optical scattering force <**F**(*x*, *y*)> was numerically simulated by using the aforementioned FDTD method. The time-averaged optical scattering force <**F**(*x*, *y*)> of the hybrid vortex mode in the azo-polymer is given by

(7) $$\left\langle F\right\rangle \propto \left[\chi_{r}\text{Im}\left(E\times B^{*}\right)+\chi_{i}\text{Re}\left(E\times B^{*}\right)\right]$$

where *χ* (=*χ*_*r*_ + *iχ*_*i*_) is the complex electric susceptibility of the azo-polymer (this value depends strongly on compounds, polymer length, polymer density, etc., and it is difficult to uniquely determine this value).

**Figure 6:**
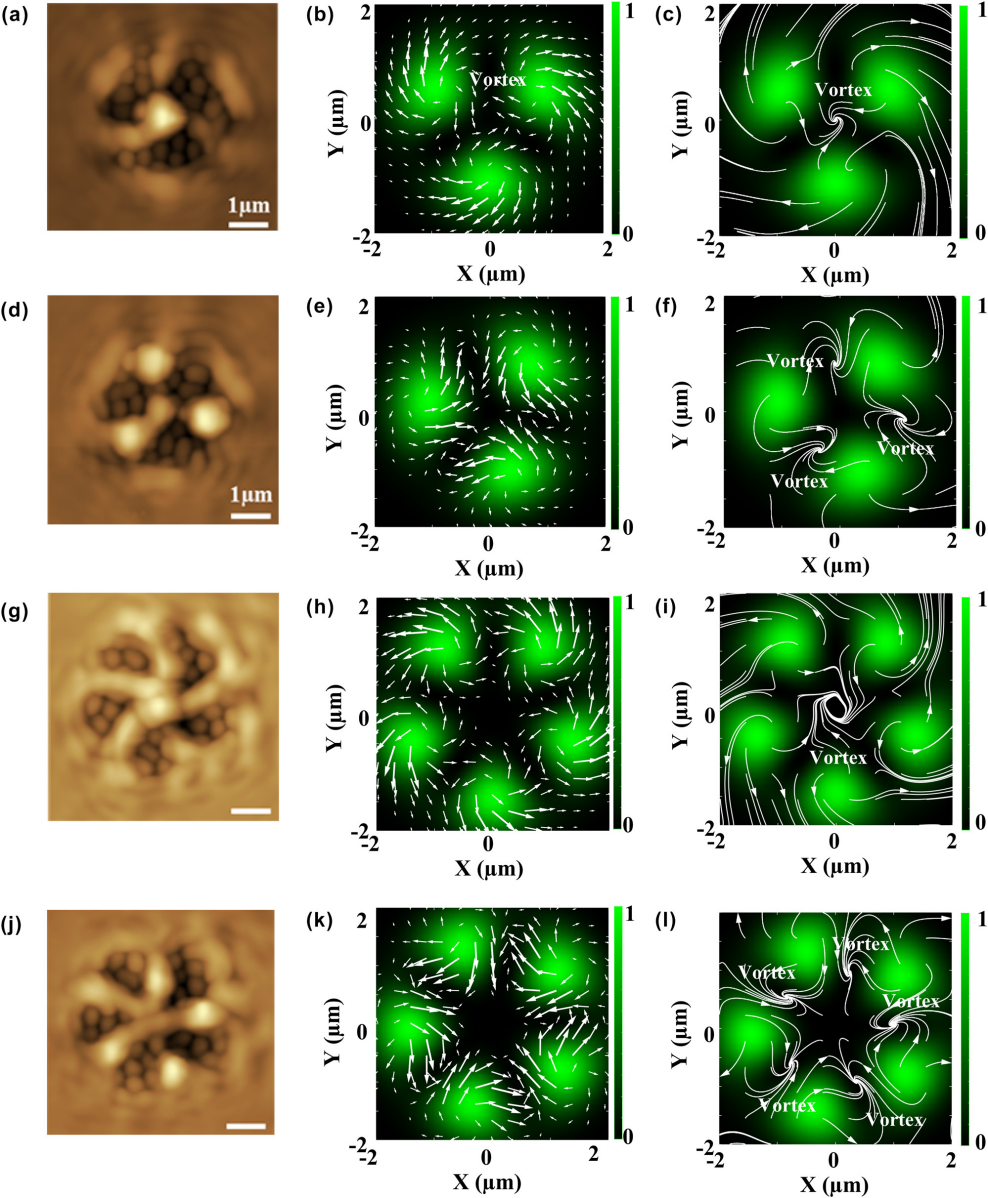
Images showing the experimentally fabricated surface relief structures (a, d, g, j), and corresponding theoretical plots of the time-averaged transverse scattering force (b, e, h, k) and mass-transport streamlines (connecting transverse scattering force vectors) (c, f, i, l) created by left-handed (*ℓ* + *ℓ′* = 1) 2^nd^ order and right-handed (*ℓ* + *ℓ′* = −1) 3^rd^ order hybrid vortex modes *ℓ* and *s* possess the same sign and *ℓ*′ and *s* possess the opposite sign. Size of simulation graphs is 4 × 4 µm. Scale bar in optical field intensities indicates brightness from maximum (whitish green) to minimum (black). Transverse scattering force vectors and mass-transport streamlines are shown by white arrows.

When *ℓ* had the same sign as *s* (*ℓ*′ had the opposite sign as *s*), the non-degenerate hybrid vortex mode produces a counterclockwise (or clockwise) inward vortex force from 3 (or 5) petal-like bright spots which are directed towards the central dark spot (see scattering force vector plots in Figure 6(b) and (h)). This results in the confinement of azo-polymers in the central dark spot and the formation of chiral surface relief structures with a central core and 3 (or 5) curved arms (see mass-transport streamline plots in Figure 6(c) and (i)). The vortex force is reversed towards the clockwise direction outside the hybrid vortex mode field, and it directs the azo-polymers in the clockwise direction, thus allowing the production of the outer rim in the surface relief structure.

When *ℓ* had the opposite sign as *s* (*ℓ*′ had the same sign as *s*), the inward vortex force, which is directed towards the central dark spot, disappears, and instead, the vortex force directs the azo-polymer towards the multiple dark nodes (see the scattering force vector plots in Figure 6(e) and (k)). This results in the formation of surface relief structures with straight arms and 3 (or 5) fists (see mass-transport streamline plots in Figure 6(f) and (l)).

These simulations demonstrate how non-degenerate hybrid vortex modes can drive the mass-transport of azo-polymers to form fist-like protrusions and therefore visualization of local vortices in fabricated surface relief structures, though the sharp bends of arms around multiple vortices seen in simulated mass-transport streamlines for *ℓ* having the opposite sign of *s* are not observed because of the limitation of the spatial resolution of AFM on structure with submicron-scale steep change in height. Alternative way is to imprint the surface relief on a glass plate by a nanoimprinting technique as mentioned in our previous publication [20], and the sharp bends of arms around multiple vortices will then be possible to be observed by employing a scanning electron microscope (SEM) in combination with metal film coating.

**Figure 7:**
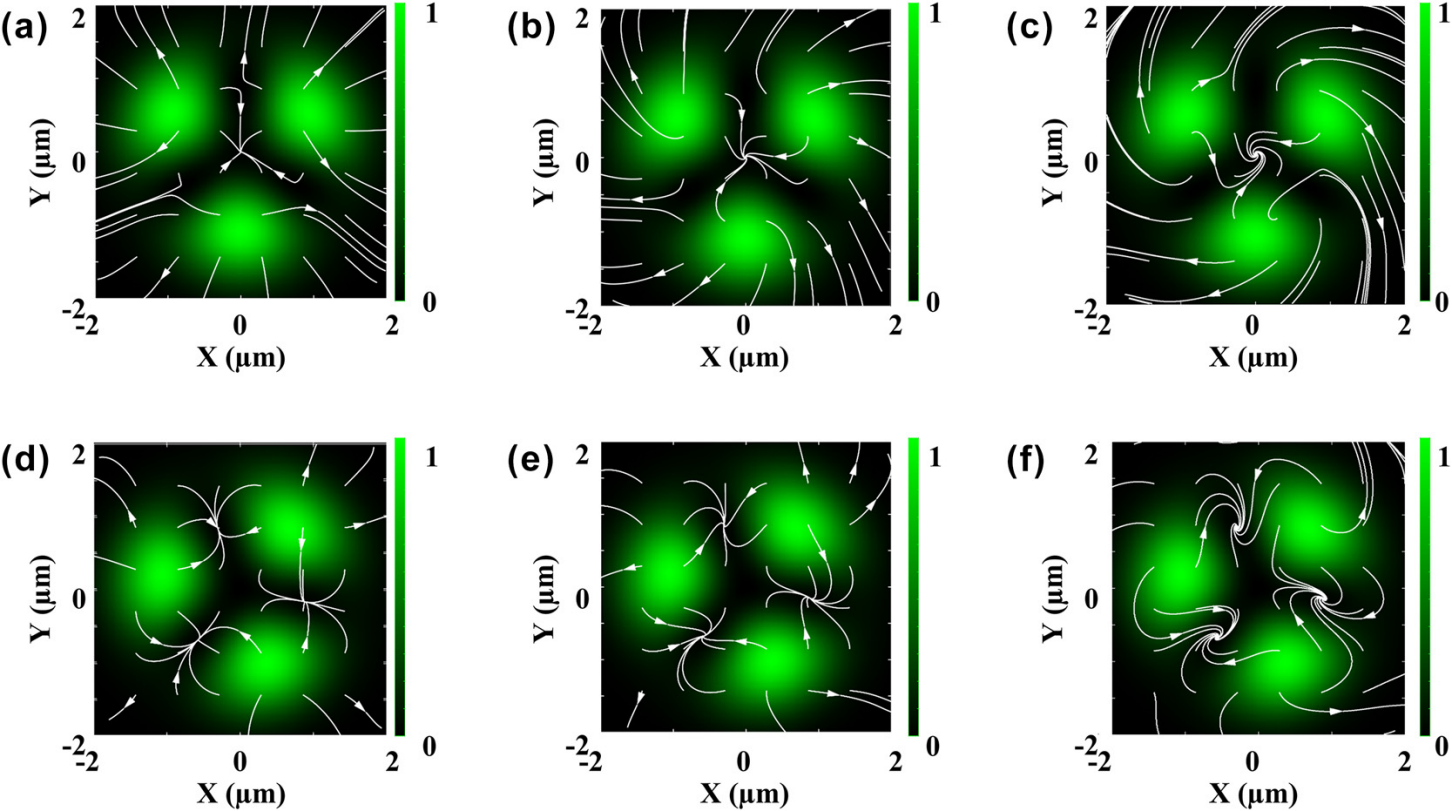
Images showing the simulated mass-transport streamlines (connecting transverse scattering force vectors) created by left-handed hybrid vortex mode (*ℓ* + *ℓ′* = 1) when *ℓ* and *s* possess the same sign (upper) and *ℓ* and *s* possess the opposite sign (lower) at various *χ*_*i*_/*χ*_*r*_. (a, d) *χ*_*i*_/*χ*_*r*_ = 0.1, (b, e) *χ*_*i*_/*χ*_*r*_ = 0.3, (c, f) *χ*_*i*_/*χ*_*r*_ = 1, respectively. Size of simulation graphs is 4 × 4 µm. Scale bar in optical field intensities indicates brightness from maximum (whitish green) to minimum (black). Mass-transport streamlines are shown by white arrows.

Note that a ratio *χ*_*i*_/*χ*_*r*_ was then assumed to be 1 in all simulations shown in Figure 6. In fact, numerical simulations were performed by varying the ratio *χ*_*i*_/*χ*_*r*_ in the range of 0.1–1. As shown in Figure 7, the vortex positions remain unchanged regardless of *χ*_*i*_/*χ*_*r*_, while the vortex forces then increase significantly as the increase of the absorption of azo-polymers (the increase of *χ*_*i*_). Though the current configuration is limited to non-degenerate hybrid vortex modes with $\left|\ell \right|$ − $\left|\ell^{\prime }\right|$ = 1, it will be extended as a universal approach to directly imprint more complex vortex structures (such as vortex lattices) of non-degenerate hybrid vortex modes with $\left|\ell \right|$ − $\left|\ell^{\prime }\right|$ > 1 (Figure S1).

## 4 Conclusions

We have demonstrated the formation of surface relief structures by using non-degenerate hybrid vortex beams, formed by the coherent superposition of two LG modes with different OAM indices, and with SAM. The fabricated surface relief structures possess fist-like protrusions, which are a result of the direct imprint of local vortices in the non-degenerate hybrid vortex modes. Interestingly, when *ℓ* and *s* possess the same sign (*ℓ*′ and *s* possess the opposite sign), the surface relief structures are twisted. Also, they exhibit $\left|\ell -\ell^{\prime }\right|$ curved arms, reflecting the trajectory of mass transported azo-polymers from $\left|\ell -\ell^{\prime }\right|$ petal-like bright spots, associated with the central vortex field. The handedness of the fabricated surface relief structures could then be reversed by simply inverting the sign of all indices, *ℓ*, *ℓ*′, and *s*. Also, note that the azimuthal orientation angle of the fabricated surface relief structures can be controlled by properly setting the relative phase between the two superposed LG modes with different OAM indices. When *ℓ* and *s* possess the opposite sign (*ℓ*′ and *s* possess the same sign), the inward vortex force directed towards the central dark spot disappears, and instead, the surface relief structures with 3 (or 5) fists were formed, demonstrating the visualization of local vortices.

Such demonstrations, in which spatially localized optical vortices of the non-degenerate hybrid vortex modes are directly imprinted (and thus visualized) in materials, provide new fundamental physical insights into light–matter interactions. We anticipate that this will also contribute to a deeper understanding of the formation of vortex lattices and vortex–antivortex pairs in condensed matter physics. Also, this work will offer a new degree of freedom in the development of rewritable engineered and integrated optical materials, including optical data storage technologies and chiral metasurface fabrications. Furthermore, it is interesting to consider the possibility of the formation of higher-order chiral structures with an odd number of arms, such as multiple-helix photopolymerized fibers, which are inherently unattainable in nature [36], [37].

## Supplementary Material

Supplementary Material Details
