# Supplementary material for: Surface relief formation with light possessing multiple vortices
Source: Nanophotonics. 2025 Nov 18;14(24):4311–22. doi: 10.1515/nanoph-2025-0387 (PMC12704484; doi:10.1515/nanoph-2025-0387)

Supplementary Material

Surface relief formation with light possessing multiple vortices

Junjie Zhao^1^, Kazuro Kizaki^1^, Atsushi Taguchi^2^, Madoka Ono^1^, Soki Hirayama^3^, Takashige Omatsu^3^*

1 Department of Applied Physics, Graduate School of Engineering, Tohoku University, Sendai, Japan

2 Research Institute for Electronic Science, Hokkaido University, Sapporo, Japan

*3 Molecular Chirality Research Center, Chiba University, Chiba, Japan*

S.1 Surface reliefs using hybrid vortex modes with |$\mathcal{l}$| - |$\mathcal{l}^{'}$| ≠ 1

The demonstration, in which spatially localized optical vortices of the non-degenerate hybrid vortex modes are directly imprinted in materials, will be extended as a universal approach to directly imprint more complex vortex structures (such as vortex lattices) of non-degenerate hybrid vortex modes with |$\mathcal{l}$| - |$\mathcal{l}^{'}$| > 1.

We further have performed the formation of surface relief structures by using hybrid vortex beams, formed by the coherent superposition of circularly polarized ±7^th^ and ±1^st^ order LG modes with a mixture ratio of 2:1 and *s*=1. When $\mathcal{l}$ and *s* possess the opposite sign, the surface relief structures exhibited 6 (corresponding to $|\mathcal{l}$- $\mathcal{l}^{'}|)$ azimuthal fists and central core, reflecting the existence of 7 local vortices (Fig. S1(a)). In contrast, when $\mathcal{l}$ and *s* possess the same sign, the surface reliefs showed no remarkable structures (Fig. S1(b)). These demonstrations also show enhancement and reduction of Poynting momentum through constructive and destructive orbital and spin momentum coupling effects in multiple vortices.

**Figure S1** Figure S1 depicts the experimentally fabricated surface relief structures, and corresponding theoretical plots of the spatial intensity profile, time-averaged scattering force and mass-transport streamlines (connecting scattering force vectors) produced by left-handed and right-handed hybrid vortex modes with *s*=1. Size of simulation graphs is 8 x 8 µm. Scale bar in optical field intensities indicates brightness from maximum (whitish green) to minimum (black). Scattering force vectors and mass-transport streamlines are shown by arrows.


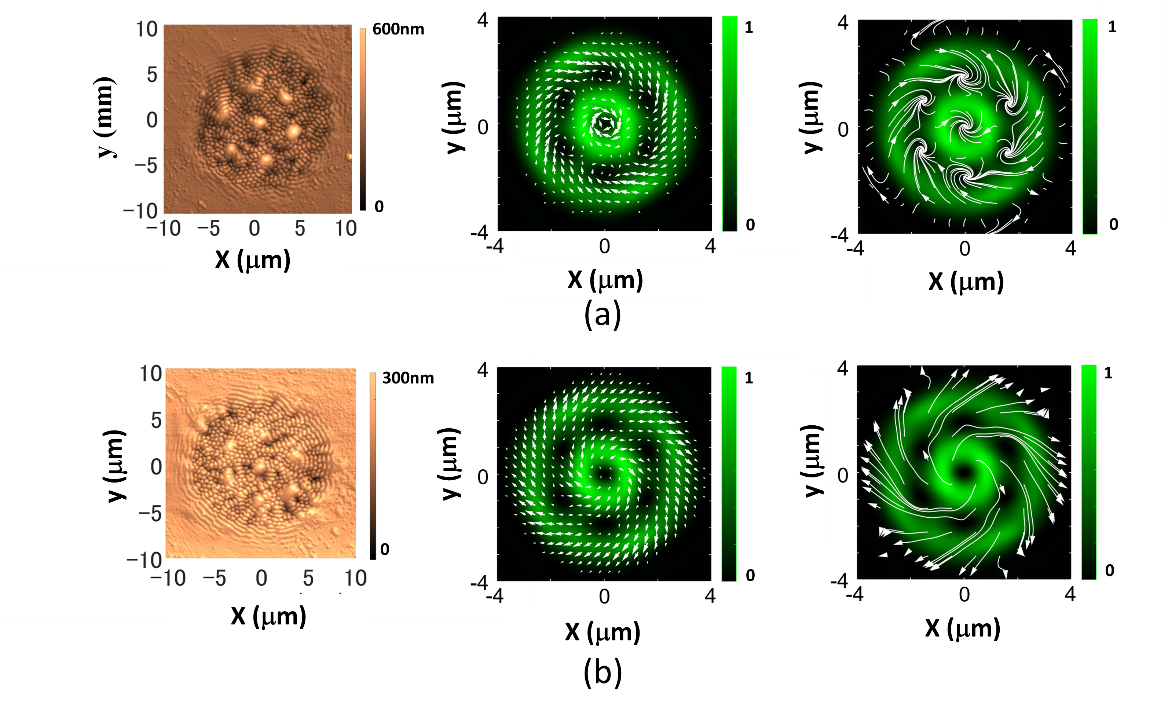

Supplement: Supplementary file 1 — Supplementary Material Details [file j_nanoph-2025-0387_suppl_001.docx]
